# Recommendations for primary care physicians to improve HPV vaccination rates during clinical encounters

**DOI:** 10.1186/1750-4732-2-10

**Published:** 2008-10-23

**Authors:** Roberto Cardarelli, Kathryn M Cardarelli

**Affiliations:** 1 Primary Care Research Institute, UNT Health Science Center at Fort Worth, 3500 Camp Bowie Blvd, Fort Worth, Texas 76107, USA; 2 Center of Community Health, UNT Health Science Center at Fort Worth, 3500 Camp Bowie Blvd, Fort Worth, Texas 76107, USA

## Abstract

The availability of the human papillomavirus (HPV) vaccine has positioned primary care physicians to play an active role in ensuring its successful implementation. However, physicians must be aware of common knowledge, attitudes, and belief barriers associated with HPV and the vaccine that are often encountered during clinical visits. This editorial provides primary care physicians an overview of these barriers and realistic recommendations utilizing the "5A's" – Awareness, Assess, Address, Acceptability, and Activate. This mnemonic can help facilitate a physician's systematic approach to increasing HPV vaccination rates during the clinical encounter.

## 

Human papillomavirus (HPV) is the most common sexually transmitted disease (STD) in the United States and the predominant cause of cervical cancer and genital warts [1,2]. In 2006, the United States Food and Drug Administration approved the first HPV vaccine for females between the ages of 9 and 26 to protect them against the 6,11,16,18 HPV strains. Phase II and III clinical trials have shown the vaccine to be efficacious in preventing cytologic abnormalities (cervical intraepithelial neoplasia I, II, and III), genital warts, and vulvar or vaginal neoplasia [3-5]. Prevention rates were high in these studies, ranging from 89–100%. However, caution is advised in interpreting these results and providing information to patients and caregivers because of the short duration of the trials (approximately 2 years). It remains unknown whether the introduction of this vaccine will prevent future cervical cancer cases and associated deaths. However, the strong biologic plausibility encourages researchers, politicians, and clinicians to implement HPV vaccination programs.

Primary care physicians (PCPs) are uniquely positioned to deliver a HPV vaccination preventive strategy to their communities since most preventive care services occur in the outpatient setting. Unfortunately, one report estimated 10% of females ages 18–26 have received at least one dose of the vaccine [6]. The purpose of this editorial is to provide recommendations to PCPs to overcome potential interpersonal and clinical barriers associated with HPV vaccination. While cost and access to care are two system barriers associated with HPV vaccination, this editorial focuses on qualitative factors that may be encountered during a clinical visit. PCPs must be aware of individual factors impeding successful HPV vaccination programs in clinics and communities. Armed with this knowledge, PCPs can mediate informational barriers and misconceptions often associated with the HPV vaccine. A shared-decision making and patient-centered approach is recommended when introducing the HPV vaccine to patients/caregivers.

Since the HPV vaccine is predominantly offered for patients of younger ages, PCPs must be willing and able to communicate with not only the patient, but, more importantly, the caregiver of the patient. Females under the age of 18 receiving the vaccination must be accompanied by a parent to provide informed consent. Hence, it is not surprising that several barriers are associated with concerns, beliefs, and knowledge deficits of the parent or caregiver.

One concern is that receiving the HPV vaccination may "condone" risky sexual behaviors among young females [7]. In reality, studies have shown that only 6–12% of caregivers are actually concerned about this issue [8-11]. Research also reports that teenage girls were no more interested in risky behavior if they were to receive the vaccination [12]. PCPs must remember to educate caregivers and patients that the vaccine does not protect them from other STDs and other strains of the HPV and that safe sex practices and screening Papanicolaou tests must always be followed. Caregivers do not see a need for the HPV vaccination if they believe their child is not sexually active [10]. In this instance, the PCP should emphasize that the ideal time to administer the vaccine is prior to sexual initiation. The patient/caregiver must be aware the HPV vaccine is not currently approved for males. Although this may change as more evidence accrues, the principle of prevention must be emphasized with patients and caregivers.

A decision to not receive the HPV vaccine may also be due to religious/spiritual factors [13]. Regardless, the PCP should be aware that patients and caregivers are accepting of vaccination and the PCP should still inform them about the availability of the vaccine and its benefits and risks (e.g., pain, discomfort, allergic reaction). In fact, risks are a frequent concern that patients express as they want to be educated about the vaccine's safety [14]. The trials have found the vaccine to be safe, with pain and discomfort at the injection site being the most common complaints [5]. Long term consequences are undetermined due to the recent introduction of the vaccine. Patients and caregivers also want to know if the vaccine actually works and PCPs must be prepared to provide this information [5]. As stated previously, although the vaccine has been shown to prevent 89–100% of cytologic abnormalities, genital warts, and vulvar or vaginal neoplasia several years after the completion of the series [3-5], patients and caregivers must also be informed that long term outcomes remain unknown.

While PCPs routinely educate patients during clinical visits, it becomes even more important when discussing HPV and the vaccination. Studies have shown that the public is largely unaware of HPV, its consequences, and the availability of a vaccine [15]. This is especially true for different racial/ethnic groups and PCPs must be proactive in establishing a successful HPV vaccination program in their clinic. A major barrier to successful vaccination programs is that teenagers do not frequently seek preventive visits [16]. Therefore, PCPs must offer the vaccination during other office visits, such as acute visits. It is important to inform patients/caregivers that the HPV vaccination is a 3 injection series (0, 2, 6 months) and that compliance is extremely important for the vaccine to take effect. Patients and caregivers must take responsibility for following up for remaining injections. However, automatic reminders or other system measures are usually necessary to achieve high completion rates. PCPs should consider utilizing vaccination flow sheets, call-back reminders, or other interventions that are simple, effective, and sustainable.

The most important factor in receiving the HPV vaccination is for the PCP to discuss and offer it to eligible patients [17,18]. As mentioned earlier, PCPs need to take an active role for HPV vaccination rates to increase. Waiting for patients and caregivers to inquire about the HPV vaccine will only have a negligible impact on the rates. To assist PCPs in becoming active leaders in successful clinical HPV vaccination programs, the authors recommend remembering the "5 A's":

**A**wareness – Ask patients/caregivers what they know about HPV, its causes, and their knowledge about an available vaccine. PCPs must be vigilant in initiating discussions and offering the vaccine to eligible patients.

**A**ssess – Inquire about any concerns related to the vaccine's safety, side-effects, efficacy and effectiveness, and potential changes in risky sexual behaviors.

**A**ddress – Provide explanations and evidence-based information using easy-to-understand language. Be proactive in providing reassuring information even if not asked or expressed by patients/caregivers.

**A**cceptability – Once the PCP assesses and addresses any issues or concerns expressed by the patient/caregiver, the PCP needs to determine whether the HPV vaccine is acceptable to the patient/caregiver in the context of cost, insurance coverage, religious/spiritual determinants, or any other factors.

**A**ctivate – Once the PCP and the patient/caregiver have jointly determined an acceptable plan, the first of 3 injections should be given. The PCP must emphasize the importance of completing the 3 dose vaccination series and, if possible, develop a plan to help themself and the patient/caregiver remember follow-up dates.

Effective office-based HPV vaccination programs involve an integrated team approach that is activated by both physicians and patients/caregivers. Successful national childhood vaccination programs should serve as models for HPV vaccination programs. Cost, attitudes, beliefs, and targeted demographics related to the HPV vaccine make the challenges unique. However, dedicated efforts by physicians and staff can be effective in overcoming such challenges.

## Competing interests

The authors declare that they have no competing interests.

## Authors' contributions

RC and KMC conceived of the manuscript and drafted all aspects of the manuscript. All authors read and approved the final manuscript
